# Adaptation and validation of the Indonesian version of the early feeding skill assessment tool for low birth weight infants

**DOI:** 10.1038/s41598-025-15342-9

**Published:** 2025-08-23

**Authors:** Suni Hariati, Erfina Erfina, Andi Dwi Bahagia Febriani, Ema Alasiry, Asriaty Asriaty, Haerani Haerani

**Affiliations:** 1 Pediatrics Nursing Department, Faculty of Nursing, Hasanuddin University, Makassar City, 90245 Indonesia; 2 Present Address: Maternity Nursing Department, Faculty of Nursing, Hasanuddin University, Makassar City, 90245 Indonesia; 3 Present Address: Department of Pediatrics, Faculty of Medicine, Hasanuddin University, Makassar City, 90245 Indonesia; 4 Present Address: Neonatal Intensive Care Unit, Wahidin Sudirohusodo Hospital, Makassar City, 90245 Indonesia; 5 Present Address: Pediatric Nursing Department, Panrita Husada Health School, Bulukumba District, 92561 Indonesia

**Keywords:** Low birth weight, Feeding behavior, Psychometrics, Premature infants, Instruments, Paediatrics, Neonatology, Preterm birth

## Abstract

Initiating oral feeding at inappropriate times can cause stress and complications for Low Birth Weight (LBW) infants. It is essential to assess the infant’s readiness for oral feeding to minimize stress and support the transition to full oral feeding. An objective assessment tool is necessary to improve the accuracy of determining the right time to start oral feedings. This study aimed to translate, adapt, and psychometrically validate the original English version of the Early Feeding Skill Assessment Tool (EFS) into the Indonesian language. The EFS questionnaire, an observation instrument with 19 items, uses a 3-option scoring structure: not yet evident, emerging, or consistently observed. The following EFS adaptation phases: (1) forward translation; (2) synthesis of forward translation; (3) back translation; (4) harmonization of the original and back translation; (4) pre-testing with the expert committee for content and relevance; (5) pre-testing with target user population for clarity; and (6) field testing. The translation was carried out by a certified translator in conjunction with a bilingual health professional. Seven neonatal experts participated in the pre-testing phase, while six nurses engaged with the target population for further evaluation. A total of 128 observations were conducted on infants exhibiting stable hemodynamics during the field testing phase. The two forward translations differed in 12 items, leading to expert discussions and consensus on the translation. The pre-testing among seven expert for content validity showed The EFS I-CVI and S-CVI/ave were 1 for all questions, indicating full expert agreement. The pre-testing among six nurses for face validity showed clear instructions (93%), with question agreement ranging from 60 to 100%. Six items had < 80% agreement and were rewritten as suggest from nurses. In field testing from 128 observations among 52 LBW infants revealed 19 items were valid and their reliability was 0.918. The 19-item EFS Indonesian version is valid and reliable for LBW infants. It can be used to implement as an assessment for a common language for infant feeding skills among interprofessional teams, contributing to infant feeding success.

## Introduction

Low birth weight (LBW) is a weight at birth less than 2500 grams regardless of gestational age, which is a global burden causing neonatal mortality and various health issues, both short-term and long-term^1,2^. LBW, especially with preterm infants, are frequently faced with complications from breathing difficulties, feeding challenges, poor temperature regulation, and a high risk of infection^2,3^. These issues lead to unstable vital signs in infants and difficulties with oral feeding, particularly in those born before 33 weeks of gestation^4^. These feeding difficulties arise from delays in nervous system development, leading to weak sucking muscles and unstable oral function. As a result, preterm infants are more susceptible to feeding issues, nutritional deficiencies, and growth delays^5,6^. Beyond the maturation of the central nervous system, the development of cardiorespiratory and oral muscular systems are also crucial determinants of oral feeding difficulties in premature infants^7^. Consequently, infants experience oral feeding difficulties due to the maturation of organs in relation to their gestational age at birth.

Oral feeding demands the integrated coordination of nutritive sucking, swallowing, and breathing^8^. The development of oral feeding starts around 28–33 weeks of gestation with the maturation of sucking-swallowing coordination, initially involving non-nutritive sucking (NNS) and advancing to a coordinated sucking-swallowing-breathing pattern for nutritive sucking (NS) by 32–34 weeks of gestation, marked by enhanced rhythmicity, amplitude, and volume^9,10^. The majority of preterm infants often experience difficulties in starting to suck, including irregular, weak, and ineffective sucking patterns. Preterm infants also struggle to coordinate the suck-swallow-breathe mechanism, leading to quick fatigue during oral feeding^11^. Starting oral feeding in a physiologically unstable or developmentally immature infant may result in complications such as fluid management issues, aspiration, distress, unstable heart rate, hypoxia, increased energy use, poor weight gain, and potential failure to thrive^12^. Another impact is choking, difficulty breathing, or unstable vital signs (e.g., decreased blood oxygen levels; SpO2 < 90%) during feeding for more than 5 minutes^13^. Conversely, a delay in achieving full oral feedings can delay hospital discharge and significantly increase the cost of care^12^. This feeding problem is also a common issue for infants at home^14^ and impacts hospital readmission after being discharged from the NICU^12,15^.

Initiating oral feeding at inappropriate times can result in stress that contributes to problematic infant feeding habits and hinders the development of proper feeding skills^12,16^. Assessing the infant’s readiness for oral feeding is crucial to minimize stress and support the achievement of full oral feeding^12,16–18^. Traditionally, assessing a preterm infant’s readiness for oral feeding was based solely on the required intake volume, without considering the infant’s readiness behaviors^16^. A skilled and attentive caregiver is crucial to ensuring a positive feeding experience, maximizing intake, minimizing stress, evaluating feeding performance, and providing timely interventions based on infants’ physiological and behavioral readiness^12,15,16^. The nurses play a pivotal role in initiating lactation as early as possible^19^. Assessment tools are crucial for objectively evaluating preterm infants’ oral feeding skills before and during feeding^20^. Using instruments that enable this assessment during breast or bottle-feeding, including formal readiness screening instruments, improves the accuracy of determining the appropriate time to initiate oral feedings^12,20,21^.

We conducted a literature review to find an objectively structured tool for assessing readiness for preterm oral feeding, which has undergone appropriate validation studies. Our search revealed that several instruments have been developed to objectively assess preterm infants’ feeding readiness^12,22^. We identified five instruments that assess oral feeding skills for preterm infants, while the instruments that are designed to evaluate oral breastfeeding, such as the premature oral feeding readiness assessment scale (POFRAS)^4,23,24^, others were designed to evaluate only oral bottle feeding (Preterm Infant Nipple Feeding Readiness Scale (PINFRS)^12^. The instrument that evaluates both during breast or bottle-feeding was the neonatal oral-motor assessment scale (NOMAS)^25^, the Oral Feeding Assessment in Premature Infants (OFEATINg)^26^, and the early feeding skill assessment (EFS)^27,28^. An instrument that can provide comprehensive evaluations of feeding skills across different feeding methods is needed.

The 19 items EFS was chosen as the instrument that reportedly possesses good psychometric properties, good reliability, and good construct for the original English version^28^, the Spanish^29^, Iran^20^, Turkish^30^, Portuguese^21^ and Persian^31^. The EFS assessment evaluates infants’ oral feeding abilities at different stages: before, during, and after feeding^28^. It tracks their skills from the initial oral feeding up to 52 weeks post-conceptional age and offers detailed insights into strengths, minor concerns, and significant issues through its sub-scales^20,28^. In Indonesia, nurses assess infants’ readiness for oral feeding by considering the infant’s general condition, gestational age, weight, breathing patterns, gastrointestinal tract function (gastric residue, intestinal sounds, the presence or absence of vomiting, and abdominal distension), physiological stability, and observing feeding cues, without using an objective tool assessment^32^. Hence, there is a need for an objective and effective psychometric oral feeding assessment tool to translate, cross-culturally adapt and conduct initial psychometric testing in Indonesia.

## Method

### Design

**Fig. 1:**
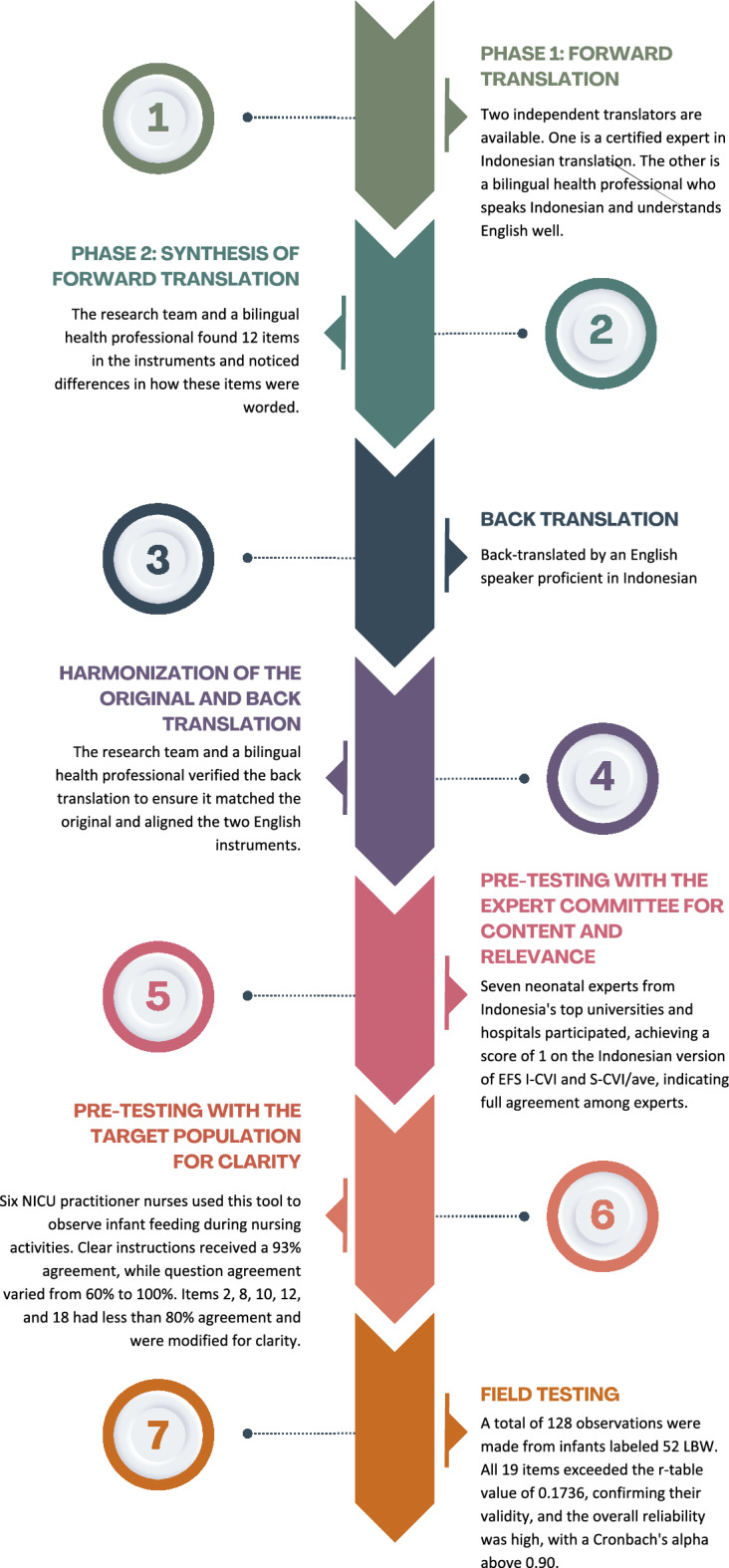
The original EFS adaptation process to Indonesian version.

The study employed cross-cultural adaptation to ensure the conceptual equivalence of the 19-item EFS scale in "the Indonesian Language" with the original English version^33–36^. The adaptation process followed the methods outlined by Cruchinho et al.^36^ and Gjersing et al.^34^. The adaptation involved the following phases: (1) forward translation; (2) synthesis of the forward translation; (3) back translation; (4) harmonization of the original and back translations; (4) pre-testing with an expert committee for content validity and relevance; (5) pre-testing with the target population for clarity; and (6) field testing. The entire adaptation process is illustrated in Fig. 1. The study was conducted from May to October 2024.

### Instruments

The EFS observational instrument was first developed by Thoyre et al. in 2005, and the latest version, a 19-item EFS, was published in 2018. The EFS evaluates an infant’s oral feeding skills and identifies the highest skill level they can demonstrate^27,28^. It can be used from the initiation of oral feeding through the maturation of oral feeding skills^27,28^. The English version of the assessment tool includes 19 items divided into five subscales: respiratory regulation (items 1-5), oral-motor function (items 6-9), swallowing coordination (items 10-13), engagement (items 14-15), and physiologic stability (items 16-19). The EFS includes two types of items related to skill development: (1) skill items that directly assess the skill, and (2) indicators of skill deficits or problematic symptoms that indirectly evaluate the level of skill^27,28,37^. Each of the 19 items is scored as 3, 2, or 1. For items (1, 2, 3, 4, 6, 7, 14, 15, 18, and 19), a score of 3 is given when the skill is consistently observed; a score of 2 is assigned if the infant demonstrates the mature pattern at least once; and a score of 1 is given if the skill is immature and not yet emerging. For items (5, 8, 9, 10, 11, 12, 13, 16, and 17), a score of 3 is given if the infant shows no signs of skill deficits; a score of 2 is assigned if the infant shows a lack of skill at least once; and a score of 1 is recorded if the infant demonstrates a lack of skill more than once or has more severe issues problems. The total scores range from 19 to 57, with higher scores indicating higher skill levels^37^.

The original version of EFS has demonstrated good validity and reliability, with internal consistency ranging from 0.70 to 0.95^28^. EFS has also been validated in other languages: the Spanish version exhibited good internal consistency with a value of 0.751^29^, the Iranian version showed good inter- and intra-rater reliability with ICC values ranging from 0.77 to 0.95^20^; the Turkish version performed excellently with a Cronbach’s alpha of 0.95^30^; the Portuguese version presented adequate factorial validity with a CFI value of 0.903^21^; and the Persian version demonstrated good reliability with a Cronbach’s alpha of 0.88^31^.

### Forward translation & synthesis of forward translation

The EFS cross-cultural adaptation process begins by obtaining permission from the instrument developer to use and adapt it for the Indonesian version, which is done by emailing the first author, Suzanne Thoyre, who has granted permission. The translation process starts with translating the English version into Indonesian. Two independent translators carry out this task: one is a certified Indonesian language expert, and the other is a bilingual health professional fluent in Indonesian with a strong understanding of English. This process yields two forward translations.

The next step involved synthesizing the two forward translations with input from a committee panel, which included the researcher’s team and the bilingual health professional. The committee identified 12 instrument items and discrepancies in wording across the instruments. These discrepancies involve sentences (such as "length of the sucking burst," "organizes long sucking bursts," "1 compression-only sucking burst," "loss of muscle tone/energy," "sucks with strong suction," "gulping or effortful hard swallows," "loss of milk at lips," and "high-pitched ‘yelping’ sound") and phrases (like "gurgling/rattle sounds," "sustains," "maintain," "sucks too long," "strong suction," "distress cues," "the sucking burst," "work of breathing," and "drops tongue”). The committee panel reached a consensus to synthesize the forward translation using Indonesian grammar and health terms used in Indonesia. The process generated an initial translated version of EFS.

### Back translation & harmonization of the original and back translation

The initial translated version of EFS was then back-translated by an English speaker proficient in Indonesian. A back translation was conducted to verify the accuracy of the forward translation^36^. The back translation was reviewed by the research team and a bilingual health professional to ensure it matched the original version and facilitated harmonization of the two English instruments. This process helps identify comparable idiomatic expressions within the “Indonesian culture”.

### Pre-testing procedure with expert and target population

The pre-testing procedure, conducted to identify potential issues affecting the reliability and validity of the translated instrument, involved evaluating the clarity and relevance of the instrument’s items^36^. This is the initial method for establishing validity in this study, which involves a logical evaluation of content validity and face validity.

In these steps, researchers evaluate the appropriateness of the translated EFS instrument before deploying it in the field test. Face validity served as the initial pre-test for clarity, involving NICU practitioner nurses using this tool to observe infant feeding during their nursing activities. Six nurses from three hospitals participated in this step. Each has over four years of experience in the NICU. Nearly all of them (n=5) have bachelor’s degrees in nursing, and one of them holds a master’s degree. The NICU practitioner was interviewed to assess their understanding, acceptability, and clarity of the EFS instrument. A group cognitive debriefing approach was used in this section. Participants collaboratively reviewed the instructions for the instrument along with each of the 19 observation items. They rated each item on a dichotomous scale of "clear” or “unclear". They provided suggestions for revisions as needed to enhance the wording for items marked as “unclear” or requiring revision. The session lasted approximately 90 minutes and followed a structured guide.

To ensure a comprehensive evaluation of the instrument’s relevance, it is recommended to conduct content validation with 5 to 10 experts^35,36^. Seven neonatal experts from leading universities and hospitals in Indonesia participated in the second pre-test to assess the clarity and relevance of the Indonesian EFS instrument. They were practitioners in neonatal nursing and experts in the field. The experts have the following educational backgrounds: three hold doctorates in nursing, two have master’s degrees in nursing, and two are specialists in pediatrics or maternity nursing. The experts assessed relevance and content validity using a scale: 1 = not relevant, 2 = cannot determine relevance, 3 = relevant but requiring minor revision, and 4 = fully relevant. The expert panel’s opinions were divided into two groups: disagree (scores 1 & 2) and agree (scores 3 & 4).

To reach a decision at this stage, conducting a statistical analysis is essential to assess the reviewers’ consistency and accuracy^36^. Item-level content validity (I-CVI) and scale-level average CVI (S-CVI/Ave) were calculated to assess the content validity of the adapted version of the EFS instrument^33,35,36^. An instrument with excellent content validity should have I-CVIs of at least 0.78 and S-CVI/Ave of at least 0.90. Any I-CVI near 0.78 must be revised, and items with low I-CVI scores must be excluded^35,36^.

### Field testing

The field testing consists of preparing the pre-final version for data collection in the target population and conducting the actual data collection^36^. The preparation involved making decisions on creating the instrument form and guidelines, training the observers, and calculating the minimum sample size. The instrument form and its guidelines have been carefully designed to enhance the accuracy and consistency of measurements during data collection.

Observers’ preparation was conducted to enhance their competence and minimize errors during data collection. Five observers were recruited for this study and trained to use the Indonesian version of the EFS instruments. The five observers hold bachelor’s degrees in nursing and have over four years of experience in the NICU. This study was carried out with five observers, and a consistency test was performed among the observers to minimize variation between two or more raters measuring the same group^38^. The observers and researchers assess the same infant to gauge their comprehension of the instruments. The analysis of data using intraclass correlation coefficients (ICC) measures interrater reliability, which is essential for clinical assessments to ensure confidence in measurements or to draw rational conclusions^38^. The ICC value calculated in this study is 0.937, indicating excellent reliability.

The cross-sectional study was conducted in three general NICU hospitals that serve as referral centers in eastern Indonesia. Each hospital has fewer than 24 beds, which limits the number of eligible infants. Therefore, multiple hospitals were selected to reach the target sample size efficiently. The initial psychometric testing was carried out from July to September 2024. Participants in this study were preterm infants in the neonatal intensive care unit (NICU) who met the following inclusion criteria: gestational age of 24 weeks or more, birth weight under 2500 grams, and stable condition. The exclusion criteria included the use of a ventilator or continuous positive airway pressure (CPAP), and infants with labio-palato schiziz, esophageal atresia, intraventricular hemorrhage, central nervous system and orofacial malformations, chromosomopathies, or bronchopulmonary dysplasia. The age at which observation occurred varied depending on when the infant met the criteria, leading to different ages among participants at the time of observation.

The appropriate sample size for psychometric testing depends on the validity method used. In this section, the validity of this study will be determined based on empirical evidence from statistical analysis (criterion-related validity). The validity coefficient, which assesses the accuracy of the measure, is determined by correlating the criterion with the instrument’s scores using Pearson’s r correlation coefficient^39^. The minimum sample size for this method is 29 samples, but it is also recommended to use a larger sample, as this will enhance the accuracy of the estimates^40^. Other considerations, the sample size for field testing was determined based on an item-to-subject ratio of at least 5:1.^41^

In this study, we analyzed a sample with a 6:1 ratio, by conducting 128 observations of infant feeding involving 52 LBW infants. Among these, 28 infants were observed over two consecutive days, and 24 over three consecutive days. The total number of observation days depended on the length of hospital stay after infants met the inclusion and exclusion criteria, continuing until their discharge. Infants who remained in care for more than three days after meeting the requirements were observed three times. Five trained observers participated in data collection. The same observer assessed each infant on all observation days to ensure consistency.

The study utilized purposive sampling across three hospitals. Data collection occurred after informing parents about the study’s objectives, procedures, confidentiality, and their right to withdraw at any time.

The psychometric data were examined using descriptive statistics, Pearson’s r correlation coefficient to evaluate validity, and measures of internal consistency reliability. Pearson’s correlation coefficient (r) evaluates the relationship between respondents’ item scores and their total scores. In analyzing the results, if the calculated r-value exceeds the r-table value or if the significance value is less than 0.05, then the items in the instrument are considered valid. In our analysis using the product-moment table for 58 respondents, the r value was determined to be 0.2542. Internal consistency reliability is measured using Cronbach’s alpha coefficient. The interpretation categories are as follows: excellent (α ≥ 0.90), good (α = 0.80–0.90), acceptable (α = 0.70–0.80), questionable (α = 0.60–0.70), poor (α = 0.50–0.60), and unacceptable (α < 0.50)^42^.

### Ethical consideration

The Ethical Review Board of the Faculty of Nursing at Hasanuddin University, Indonesia, officially approved the study. All methods used in this research strictly adhered to relevant guidelines and regulations. All parents of the LBW infants provided written informed consent before data collection started. The parents are the legal guardians of the LBW infants. Enumerators collected all questionnaires anonymously. The data are securely stored and will only be used for research purposes.

## Result

### Content validity

The Indonesian version of EFS I-CVI and S-CVI/ave both scored 1 for all questions, indicating full expert agreement. The experts also suggested terminology, such as consistently using the term “sucking” for all items in the Indonesian version. Change the bpm in item 19 to the Indonesian term “x/minute” and use the oxygen saturation standard from the Indonesian NICU room, which is over 90% instead of 85%.

### Face validity

The inter-rater agreement was calculated based on the six nurses’ responses, revealing clear instructions (93% agreement) and question agreement ranging from 60 to 100%. Six items (items 2, 8, 10, 12, and 18) showed less than 80% agreement and were modified for language accordingly. They recommended changing the Indonesian translation to “the length of the suck burst”, such as “lama hisapan” to “durasi hisapan” in item 2, and the phrase “strong suction” in item 8, such as "daya hisap kuat" to “hisapan kuat”. They also mentioned not understanding the term "gurgling/rattle sounds created by fluid in the nose or pharynx" in item 10 and the term "High-pitched ‘yelping’ sound" in item 12. They suggested providing a more detailed explanation before using this instrument. The final point they were unclear about was item 18. They suggested using the oxygen saturation standard from the Indonesian NICU room, which is over 90% instead of 85%. The instrument was revised based on language suggestions and underwent pre-testing for relevance among neonatal nursing experts.

### Psychometric testing

In this phase, it was found that there were a total of 128 observations from 52 LBW infants. The majority of infants were female (59.6%). More than half of them were delivered via Cesarean section (53.8%). Among the LBW infants, the average gestational age was 33.46 weeks, and 76,9% of them were born prematurely. The average birth weight was 1848.75 grams, and 78.8% were classified as LBW. LBW infants had APGAR scores ranging from 1 to 9 in the first minute and from 7 to 10 in the fifth minute. For more detailed results, please refer to Table 1.

**Table 1:** Demographic and clinical characteristics of infant.

Variable	n (%)	Mean ± SD	Min–Max
Infant sex			
Male	21(40.4)		
Female	31(59.6)		
Type of birth			
Cesarean	28(53.8)		
Normal birth	24(46.2)		
Primary Associated conditions			
No	36(69.2)		
Yes	16(30.8)		
Gestational ages (Weeks)		33.46(3.47)	24–39
Preterm	40(76.9)		
Aterm	12(23.1)		
Birth Weight (grams)		1848.75(450.36)	765–2490
ELBW	3(5.8)		
VLBW	8(15.4)		
LBW	41(78.8)		
APGAR Score (First minute)		7.42(1.55)	1–9
APGAR Score (Fifth minute)		8.83(.96)	7–10

Table 2 displays the r-value for each item to each sub-scale and the total scale for the Indonesian version of EFS. All items (19 in total) have an r-value higher than the r-table value (0.1736), indicating that they are valid items. This pattern remains consistent when analyzing the result of each item with its respective sub-scale.

**Table 2:** The r-value for each item to total score, its item to its subscales.

Number of item	Variable	Total score	Respiratory regulation	Oral motor function	Swallowing coordination	Engagement	Physiologic stability
1	Integrates breaths within the sucking burst	.724**	.681				
2	Times the length of the sucking burst to remain stable	.596	.739				
3	Organizes long sucking bursts (7 + sucks) without signs of behavioral or cardio-respiratory instability	.628	.807				
4	Each time the nipple is received, transitions to sucking without behavioral or cardio-respiratory signs of instability	.604	.702				
5	Increased work of breathing	.673	.727				
6	Promptly starts sucking once nipple is received	.757		.825			
7	Sucks with steady and strong suction	.751		.830			
8	Actively opens mouth and drops tongue to receive the nipple when lips are stroked	.731		.842			
9	Loss of milk at lips	.656		.755			
10	Gulping or effortful swallows	.751			.829		
11	Gurgling/rattle sounds created by fluid in the nose or pharynx	.696			.764		
12	High-pitched “yelping” sound as the airway re-opens after the swallow	.604			.784		
13	Coughing or choking sounds	.418			.617		
14	Energy	.749				.927	
15	State	.795				.939	
16	Color change	.628					.730
17	Stable oxygen saturation	.305					.578
18	Stable heart rate	.495					.694
19	Stress	.443					.593

*P < 0.05: using Pearson correlation analysis; r-table = .1736.

*,*P < 0.01: using Pearson correlation analysis.

The Indonesian version of EFS demonstrated excellent reliability for total items, with a Cronbach’s alpha above 0.90. The reliability for two subscales—Oral Motor Function and Engagement—was in the good range (Cronbach’s α between 0.80 and 0.90). However, the Respiratory Regulation and Swallowing subscales showed acceptable reliability (Cronbach’s α between 0.70 and 0.80), while the Physiologic Stability subscale exhibited poor reliability (Cronbach’s α between 0.50 and 0.60). For more detailed results, please see Table 3.

**Table 3:** Cronbach’s alpha coefficient for total scale and the five subscales.

Variable	Cronbach’s alpha coefficient
Total item of the Indonesian version of EFS	.918
Subscale respiratory regulation	.783
Subscale oral motor function	.830
Subscale swallowing coordination	.742
Subscale engagement	.849
Subscale physiologic stability	.545

## Discussion

Prior to initiating oral feeding in a physiologically unstable or developmentally immature infant, it is important to consider the potential risks, including fluid management issues, aspiration, distress, unstable heart rate, hypoxia, increased energy use, poor weight gain, and potential failure to thrive^12^. There is a growing need for reliable and valid tools to assess an infant’s oral feeding skills before starting oral feeding. Currently, most of nurses in Indonesia assess infants’ readiness for oral feeding without using objective assessment tools. Therefore, it is crucial to utilize highly validated and reliable tools that have been previously developed for use in Indonesia. The Indonesian EFS tool enables safer, personalized feeding decisions for preterm or developmentally immature infants, potentially improving nutritional outcomes, reducing complications, and enhancing growth and development. Some infants find oral feeding complex; therefore, EFS helps decide to postpone it and prevents negative feeding experiences that may hinder future oral feeding^28^. Hence, assessment is essential in avoiding long-term feeding issues.

Translation was the initial step before tool adaptation. This study used two independent translators for forward translation, synthesizing the two EFS forward translations and then back translation by one translator. The comparison of two EFS forward translations and the original version was conducted to achieve a consensus on ambiguity and discrepancies of words, sentences, and meanings in Indonesian culture and language^33,35^. In this process, the committee identified several discrepancies in the EFS forward translation and reached a consensus on their resolution. The interpretation of original tools can be tailored to fit the cultural context while still adhering to criteria that prioritize source accuracy, comprehensibility, cultural appropriateness, and correct terminology to inform decision-making processes^36^. Terminology adjustments and clinical parameters were also tailored to ensure the instrument aligns with local practices and needs^43^. This process influences clinical decision-making in Indonesia, where culturally misaligned tools may misinterpret infants’ readiness for feeding, leading to inadequate care. Adapting the EFS to the Indonesian context enhances its reliability in helping healthcare providers assess the preparedness of preterm infants for oral feeding, ensuring safer practices and minimizing risks. Healthcare providers require a common language to articulate the evaluation of infant feeding skills and their variations, as this can facilitate the organization of data or observations into evaluative domains that support team discussions regarding infant progress^27^

The Indonesia EFS tools demonstrated good validity, as content validity was assessed by seven neonatal experts in Indonesia, and face validity was evaluated by six neonatal nurses from the hospitals. This EFS also demonstrates good validity for adaptation in other countries, including Spain, Turkey, Portugal, and Iran^20,21,29–31^. This study is different from the study in Persian in that in content validity, they eliminated 2 items (items 12 and 15) that have low content validity; in our study, there are no items eliminated in the content validity phase^31^. The content validity in this study is in line with the study in Portugal and Turkey version that there are no elimination items in content validity^21,30^. In the Spanish version, they did not conduct content validity^29^. This phase is mentioned as pilot testing of the pre-final version to achieve cognitive debriefing^35^.

The findings align with validation studies of EFS in other countries but with some differences. For example, studies in Iran and Indonesia required modification of some EFS items for local context. Some versions, like the Portuguese and Spanish ones, had lower reliability in certain subscales, indicating a need for more sensitive measurement instruments. However, the instrument demonstrated good validity and reliability after adaptation in Turkey, highlighting the importance of user training for consistent interpretation of complex items.

The results are consistent with validation studies of EFS conducted in other countries, but there are some significant differences. For instance, a study by Kamran et al. (2021) in Iran also assessed the validity and reliability of the EFS, and the results were largely in line with our study. Zinoni et al. (2021) validated the EFS in Spain and found good validity for most items. They found satisfactory and excellent interrater reliability among observers for 57.69% of the items in the EFSA 2010 tool, a property that improved in the EFSA 2018 tool to 73.68%. They have not yet evaluated the internal consistency reliability among the infants. Other validation by Girgin et al. (2021), conducted in Turkey, found that after adaptation, the instrument demonstrated good validity and reliability. However, they emphasized the importance of training users to ensure consistent interpretation of more complex items, particularly those related to swallowing coordination and oral motor function.

Other research by Dos Santos Curado et al. (2017), who validated a modified version of EFS across three subscales for the Portuguese version, reported high composite reliability (coordination of feeding (COFO)=0.953; Cardiorespiratory Coordination and/or Dysregulation (CCD)=0.861; and Complexity of Meal-Related Feeding (CEMF)=0.951). However, CCD has lower reliability than other subscales, particularly Swallowing Coordination, suggesting a need for more sensitive instruments to assess swallowing coordination. This finding aligns with our research, which revealed that the subscales for respiratory regulation and swallowing demonstrated acceptable reliability (Cronbach’s α between 0.70 and 0.80). This study examines three hospitals with differing feeding conditions, leading to variations in external factors like temporary instability during feeding and room conditions that cannot be controlled, which may affect scoring variability. Creating an ideal environment enhances assessment feeding by ensuring the infant is fed in the quietest setting, eliminating distractions, and minimizing interruptions. It is important to devote complete attention to the infant during the feeding process^27^.

In the Indonesian version, the subscale of physiologic stability, which reflects stress, color, heart rate, and oxygen saturation, demonstrated poor reliability. This study focused solely on physiologically stable infants and employed continuous oxygen monitoring during the EFS assessment, which likely resulted in minimal data variability. On the other hand, a previous study was performed on infants 32-37 weeks of gestational age, without considering physiological stability^21,31^. This condition may explain the lower reliability. Additionally, assessing physiological stability, especially stress and color, can be subtle and subjective, even among trained observers, which may explain the lower interrater reliability seen in this subscale.

Early assessment of feeding skills is one of the crucial evaluations to minimize stress and promote the achievement of full oral feeding. This study provides a validated tool for assessing early feeding skills among LBW with good validity for each item and excellent reliability for the total items. These tools are especially important in Indonesia, where the rate of postnatal growth failure (PGF) in preterm infants is notably high at approximately 47,2% (based on the weight-for-age indicator of PGF) and this PGF rate is linked to the timely provision of adequate nutrients^44^. On the other hand, in Indonesia, the average length of stay is approximately 7.5 days, influenced by extended parenteral feeding and delayed enteral feeding^45^. Therefore, this instrument is expected to help nurses better discern infants’ readiness for oral feeding, allowing for more timely and targeted interventions. Until this study, there was no standard instrument or tool that specifically assessed early feeding skills in the Indonesian setting. Consequently, it may reduce the likelihood of postnatal growth failure and shorten hospital stays. Given that NICUs in Indonesia often face bed shortages and resource constraints, an effective tool for assessing feeding readiness can greatly enhance clinical decision-making and optimize resource utilization.

The results of this research carry important clinical implications. This validated instrument offers a way to enhance feeding assessment and intervention in Indonesia by evaluating feeding readiness early and objectively. This enables health workers to begin oral feeding at the right time, potentially reducing long-term complications. Additionally, this tool improves healthcare efficiency. In resource-constrained NICUs, such as those in Indonesia, where prolonged hospital stays often result from delayed feeding transitions, this tool is expected to facilitate timely clinical decisions, shorten hospital stays, and enhance bed turnover rates. Therefore, the Indonesian version of the EFS tool serves as a valuable clinical resource and a strategic method to address healthcare delivery challenges.

### Limitation

The study faced constraints due to the limited number of infants available from the three hospitals and the involvement of multiple facilities, which might introduce variability. Nonetheless, all observers underwent training and standardization prior to data collection to minimize inter-observer differences. However, despite this limitation, the study demonstrated good validity and reliability. Furthermore, the items that have lower skor of r-table may be more relevant to other types of infants, making further testing beneficial.

## Conclusion

The EFS Indonesian Version have accepted all items from the original version. The 19-item EFS Indonesian version, having been found to be valid and reliable for LBW infants, can be used to develop a common language for infant feeding skills among interprofessional teams. This involves using a standardized tool to assess and communicate about infant feeding skills, which can lead to more coordinated and effective care. By contributing to infant feeding success, this tool can potentially improve the overall care of LBW infants.

We recommend expanding the use of this tool throughout NICU environments in Indonesia, accompanied by training programs to promote consistent application among healthcare providers. Additionally, future studies should evaluate the tool’s effects on clinical outcomes like growth trajectories, length of stay, and breastfeeding success rates post-discharge.

## Data Availability

The translated version of the Indonesian EFS tool is available at https://zenodo.org/ with 10.5281/zenodo.16594432.

## Abbreviations

LBW: Low birth weight
EFS: Early feeding skill
NICU: Neonatal intensive care unit
I-CVI: Item-level content validity
S-CVI/Ave: Scale-level average
CPAP: Continuous positive airway pressure
NNS: Non-nutritive sucking
NS: Nutritive sucking
POFRAS: Premature oral feeding readiness assessment scale
PINFRS: Preterm infant nipple feeding readiness scale
NOMAS: The neonatal oral-motor assessment scale
OFEATINg: The oral feeding assessment in premature infants

## References

[1] Ohuma, E. O. et al. National, regional, and global estimates of preterm birth in 2020, with trends from 2010: A systematic analysis. *The Lancet.* **402**(10409), 1261–1271 (2023). 10.1016/S0140-6736(23)00878-4 37805217

[2] Cao, G., Liu, J. & Liu, M. Global, regional, and national incidence and mortality of neonatal preterm birth, 1990–2019. *JAMA Pediatr.* **176**(8), 787–796 (2022). 35639401 10.1001/jamapediatrics.2022.1622 PMC9157382

[3] World Health Organization. WHO recommendations on interventions to improve preterm birth outcomes [Internet]. World Health Organization; 2015. Available from: www.who.int/reproductivehealth 26447264

[4] Chang, Y. J. et al. Clinical validation of the preterm oral feeding readiness assessment scale in Taiwan. *J. Pediatr. Nurs.* **1**(59), e84-92 (2021). 10.1016/j.pedn.2021.02.005 33648837

[5] Aboelmagd, A. N., Mohamed, S. S. & Tawfik, A. H. Effect of sensory motor stimulation on enhancing oral feeding readiness of preterm neonates. Egypt. *J. Health Care* **13**(3), (2022).

[6] Izzaturrohmah, S. & Zubaidah, Z. Implementation of preterm infant oral motor stimulation intervention (PIOMI) on very low birth weight preterm baby. *Nurse Health J. Keperawatan* **12**(1), 20–29 (2023).

[7] Chen, G., Li, X. & Pan, R. Prefeeding interventions improve oral feeding in preterm infants. *Int. J. Pediatr. Otorhinolaryngol.* **1**(162), 111324 (2022). 10.1016/j.ijporl.2022.111324 36137472

[8] Bertoncelli, N. et al. Oral feeding competences of healthy preterm infants: A review. *Int. J. Pediatr.* **2012**, 1–5 (2012). 10.1155/2012/896257 PMC3362836 22675368

[9] Lau, C. Development of suck and swallow mechanisms in infants. *Ann. Nutr. Metab.* **20**(66), 7–14 (2015). 10.1159/000381361 PMC4530609 26226992

[10] Kamity, R., Kapavarapu, P. K. & Chandel, A. Feeding problems and long-term outcomes in preterm infants—A systematic approach to evaluation and management. *Children*. **8** (2021). 10.3390/children8121158 PMC8700416 34943354

[11] Wahyuni, L. K. et al. Factors affecting oral feeding ability in Indonesian preterm infants. *Pediatr. Rep.* **14**(2), 233–243 (2022). 35645368 10.3390/pediatric14020031 PMC9149927

[12] Gennattasio, A., Perri, E. A., Baranek, D. & Rohan, A. Oral feeding readiness assessment in premature infants. *MCN Am. J. Matern. Child Nurs.* **40**(2), 96–104 (2015). 25494013 10.1097/NMC.0000000000000115

[13] Chang, Y. J. et al. Preterm oral feeding scale to assist in deciding initial oral feeding of preterm infants in neonatal intensive care units. *Pediatr. Neonatol.* **63**(3), 269–275 (2022). 35305927 10.1016/j.pedneo.2021.12.008

[14] Hariati, S. et al. Indonesian mothers’ beliefs on caring practices at home for preterm babies after hospital discharge: A qualitative study. *J. Special. Pediatr. Nurs.* **26**(3), 12330 (2021). 10.1111/jspn.12330 33773015

[15] Lubbe, W. Clinicians guide for cue-based transition to oral feeding in preterm infants: An easy-to-use clinical guide. *J. Eval. Clin. Pract.* **24**(1), 80–88 (2018). 28251754 10.1111/jep.12721 PMC5901413

[16] Osman, A. A., Ahmed, E. S., Hassanein, F. E. S., Mohamed, H. S. & Brandon, D. Assessment of oral feeding readiness among preterm infants. *Assiut Sci. Nurs. J. Osman et al* **5**(10), 116–121 (2017).

[17] McCain, G. C. An evidence-based guideline for introducing oral feeding to healthy preterm infants. *Neonatal Netw.* **22**(5), 45–50 (2003). 14598979 10.1891/0730-0832.22.5.45

[18] Griffith, T. T., Bell, A. F., White-Traut, R., Medoff-Cooper, B. & Rankin, K. Relationship between duration of tube feeding and success of oral feeding in preterm infants. *JOGNN J. Obstet. Gynecol. Neonatal Nurs.* **47**(5), 620–631 (2018). 30040913 10.1016/j.jogn.2018.06.002

[19] Hariati, S., McKenna, L., Sutomo, R., Lusmilasari, L. & Febriani, A. D. B. Indonesian mothers of premature infants’ experiences in achieving initial motherhood independence in the neonatal unit: A qualitative study. *J. Neonatal Nurs.* **29**(2), 283–289 (2023).

[20] Kamran, F., Sagheb, S., Khatoonabadi, S. A., Ebadi, A., Faryadras, Y. & Aghajanzadeh, M. The validity and reliability of early feeding skills assessment and cue-based feeding scales for preterm infants. *Middle East J. Rehabil. Health Stud*. **8**(3) (2021).

[21] Dos Santos Curado, M. A., Maroco, J. P., Vasconcellos, T., Gouveia, L. M. & Thoyre, S. Validation of the early feeding skills assessment scale for the Portuguese population. *Revista de Enfermagem Referencia.* **4**(12), 131–142 (2017).

[22] Crowe, L., Chang, A. & Wallace, K. Instruments for assessing readiness to commence suck feeds in preterm infants: Effects on time to establish full oral feeding and duration of hospitalisation. Vol. 2016, Cochrane Database of Systematic Reviews. John Wiley and Sons Ltd (2016). 10.1002/14651858.CD005586.pub3 PMC6464358 27552522

[23] Ide Fujinaga, C., Alves de Moraes, S., Ellen Zamberlan-Amorim, N., Corrêa Castral, T., de Almeida Silva, A. & Gracinda Silvan Scochi, C. Clinical validation of the Preterm Oral Feeding Readiness Assessment Scale 1. Vol. 21, Original Article Rev. Latino-Am. Enfermagem. 2013. Available from: www.eerp.usp.br/rlae 10.1590/s0104-11692013000700018 23459901

[24] Çamur, Z. & Çetinkaya, B. The validity and reliability study of the turkish version of the preterm oral feeding readiness assessment scale (T-POFRAS). *J. Pediatr. Res.* **8**(3), 225–232 (2021).

[25] Da Costa, S. P. & Van Der Schans, C. P. The reliability of the neonatal oral-motor assessment scale. *Acta Paediatr. Int. J. Paediatr.* **97**(1), 21–26 (2008). 10.1111/j.1651-2227.2007.00577.x 18201309

[26] Alonso-Fernández, S., de Liria, C. R. G., Lluch-Canut, T., Poch-Pla, L., Perapoch-López, J. & Juvé-Udina, M. E., et al. Psychometric properties of the oral feeding assessment in premature infants scale. *Sci Rep*. **12**(1) (2022). 10.1038/s41598-022-11521-0 PMC9098432 35551222

[27] Thoyre, S. M., Shaker, C. S. & Pridham, K. F. The Early Feeding Skills Assessment for Preterm Infants. *Neonatal Netw.* **24** (2005). 10.1891/0730-0832.24.3.7 PMC2828611 15960007

[28] Thoyre, S. M., Pados, B. F., Shaker, C. S., Fuller, K. & Park, J. Psychometric properties of the early feeding skills assessment tool. *Adv. Neonatal Care* **18**(5), E13-23 (2018). 30239407 10.1097/ANC.0000000000000537

[29] Matarazzo Zinoni, M., Campos Herrero, L., González Lamuño, D. & de las Cuevas Terána, I. Translation and study of the measurement properties of the Early Feeding Skills Assessment tool in premature newborn. *Anales de Pediatría (English Edition)*. **95**(2), 72–7 (2021). 10.1016/j.anpede.2020.05.018 34246623

[30] Girgin, B. A., Gözen, D., Uslubaş, R. & Bilgin, L. The evaluation of oral feeding in preterm infants: Turkish validation of the early feeding skills assessment tool. *Turk. Arch. Pediatr.* **56**(5), 440–446 (2021). 35110111 10.5152/TurkArchPediatr.2021.21008 PMC8849411

[31] Bahrami, B., Marofi, M., Farajzadegan, Z. & Barekatain, B. Validation of the early feeding skills assessment scale for the evaluation of oral feeding in premature infants. *Iran. J. Neonatol.* **10**(2), 68–75 (2019).

[32] Astuti, D. D., Rohsiswatmo, R., Wanda, D. & Utari, D. M. Experiences of Indonesian nurses in improving preterm oral feeding readiness in special care units: A qualitative descriptive study. *Belitung Nurs J.* **9**(5), 478–488 (2023). 37901376 10.33546/bnj.2772 PMC10600710

[33] Hariati, S. et al. Translation, adaptation and psychometric validation of the Indonesian version of the Readiness for Hospital Discharge Scale for parents of low birth weight infants. *J. Pediatr. Nurs.* **1**(54), e97-104 (2020). 10.1016/j.pedn.2020.05.010 32522382

[34] Gjersing, L., Rm Caplehorn, J. & Clausen, T. Cross-cultural adaptation of research instruments: Language, setting, time and statistical considerations [Internet]. 2010. Available from: http://www.biomedcentral.com/1471-2288/10/13 10.1186/1471-2288-10-13 PMC2831007 20144247

[35] Sousa, V. D. & Rojjanasrirat, W. Translation, adaptation and validation of instruments or scales for use in cross-cultural health care research: A clear and user-friendly guideline. J. Eval. Clin. Pract. **17**, 268–274 (2011). 10.1111/j.1365-2753.2010.01434.x 20874835

[36] Cruchinho, P. et al. Translation, cross-cultural adaptation, and validation of measurement instruments: A practical guideline for novice researchers. *J. Multidiscip. Healthc.* **17**, 2701–2728 (2024). 38840704 10.2147/JMDH.S419714 PMC11151507

[37] Thoyre, S. Early Feeding Skills (EFS) Assesment Scoring Guidelines (2021).

[38] Koo, T. K. & Li, M. Y. A guideline of selecting and reporting intraclass correlation coefficients for reliability research. *J. Chiropr. Med.* **15**(2), 155–163 (2016). 27330520 10.1016/j.jcm.2016.02.012 PMC4913118

[39] Odom, L. R. & Morrow, J. R. What’s this r? A correlational approach to explaining validity, reliability and objectivity coefficients. *Meas. Phys. Educ. Exerc. Sci.* **10**(2), 137–145 (2006).

[40] Adam Bujang, M., Baharum, N., Mara, T. & Alam, S. Sample size guideline for correlation analysis. *World J. Soc. Sci. Res*. **3**(1) (2016).

[41] Hair, J. F., Black, W. C., Babin, B. J. & Anderson, R. E. *Multivariate Data Analysis* 7th edn. (Pearson Education, Inc., 2013).

[42] Tavakol, M. & Dennick, R. Making sense of cronbach’s alpha. *Int. J. Med. Educ.* **2**, 53–5. 10.5116/ijme.4dfb.8dfd PMC4205511 28029643

[43] Briere, C. E., McGrath, J., Cong, X. & Cusson, R. State of the science: A contemporary review of feeding readiness in the preterm infant. *J. Perinatal Neonatal. Nurs.* **28**(1), 51–58 (2014). 10.1097/JPN.0000000000000011 24476652

[44] Rohsiswatmo, R., Kaban, R. K., Sjahrulla, M. A. R., Hikmahrachim, H. G., Marsubrin, P. M. T. & Roeslani, R. D. et al. Defining postnatal growth failure among preterm infants in Indonesia. *Front. Nutr.* **10** (2023). 10.3389/fnut.2023.1101048 PMC10042288 36992910

[45] Prashanti, N. A., Widiatmika, K. S., Suryaningsih, P. S. & Suryawan, I. W. Risk factors affecting length of stay in preterm infants at Wangaya Regional General Hospital, Indonesia. *Bioscientia Medicina J. Biomed. Transl. Res.* **8**(1), 3892–3901 (2024).

